# INHIBITION OF APOPTOSIS EXACERBATES FATIGUE-DAMAGE TENDON INJURIES IN AN *IN VIVO* RAT MODEL

**DOI:** 10.22203/eCM.v036a04

**Published:** 2018-07-30

**Authors:** R. Bell, M.A. Robles-Harris, M. Anderson, D. Laudier, M.B. Schaffler, E.L. Flatow, N. Andarawis-Puri

**Affiliations:** 1 Sibley School of Mechanical and Aerospace Engineering, Cornell University, Ithaca, NY, USA; 2 Nancy E. and Peter C. Meinig School of Biomedical Engineering, Cornell University, Ithaca, NY, USA; 3 Hospital for Special Surgery, New York, NY, USA; 4 Leni and Peter W. May Department of Orthopedics, Icahn School of Medicine at Mount Sinai, New York, NY, USA; 5 Department of Biomedical Engineering, City College of New York, New York, NY, USA

**Keywords:** Cells, tissues, ligament, tendon, extracellular matrix collagens, connective tissues, tendon, cells/tissues-aging/apoptosis, animal models

## Abstract

Tendinopathy is a common and progressive musculoskeletal disease. Increased apoptosis is an end-stage tendinopathy manifestation, but its contribution to the pathology of the disease is unknown. A previously established *in vivo* model of fatigue-damage accumulation shows that increased apoptosis is correlated with the severity of induced tendon damage, even in early onset of the disease, supporting its implication in the pathogenesis of the disease. Consequently, this study aimed to determine: (1) whether apoptosis could be inhibited after fatigue damage and (2) whether its inhibition could lead to remodeling of the extracellular matrix (ECM) and pericellular matrix (PCM), to ultimately improve the mechanical properties of fatigue-damaged tendons. The working hypothesis was that, despite the low vascular nature of the tendon, apoptosis would be inhibited, prompting increased production of matrix proteins and restoring tendon mechanical properties. Rats received 2 or 5 d of systemic pan-caspase inhibitor (Q-VD-OPh) or dimethyl sulfoxide (DMSO) carrier control injections starting immediately prior to fatigue loading and were sacrificed at days 7 and 14 post-fatigue-loading. Systemic pan-caspase inhibition for 2 d led to a surprising increase in apoptosis, but inhibition for 5 d increased the population of live cells that could repair the fatigue damage. Further analysis of the 5 d group showed that effective inhibition led to an increased population of cells producing ECM and PCM proteins, although typically in conjunction with oxidative stress markers. Ultimately, inhibition of apoptosis led to further deterioration in mechanical properties of fatigue-damaged tendons.

## Introduction

Tendinopathy is a common and progressive musculoskeletal condition and accounts for approximately 30 % of all musculoskeletal consultations (McCormick et al., 1995). Effective therapeutics have not been developed because the mechanisms that underlie the pathology of these injuries are largely unknown. Degenerative changes observed in clinical samples of late-stage tendinopathy suggest that damage accumulation due to overuse underlies the pathology (Jarvinen et al., 1997; Kannus and Jozsa, 1991; Tallon et al., 2001). Interestingly, the onset of sub-rupture fatigue injury causes a decrease in stiffness and an increase in structural damage that is not recoverable, predisposing the tendon to accumulation of further injury, even early in the pathogenesis of the tendinopathy (Andarawis-Puri and Flatow, 2011). While the biological mechanisms that drive this impaired repair response are largely unknown, several studies implicate apoptosis, or programmed cell death, as a potential contributor to the pathology (Benson et al., 2010; Lian et al., 2007; Lundgreen et al., 2011; Tuoheti et al., 2005; Wu et al., 2011).

Late-stage tendinopathy is characterized by increased apoptotic activity in conjunction with degenerative changes (Benson et al., 2010; Lian et al., 2007; Lundgreen et al., 2011; Tuoheti et al., 2005; Wu et al., 2011). For instance, biopsies from athletes with painful patellar tendinopathy exhibit increased apoptosis and glycosaminoglycans and collagen disorganization (Lian et al., 2007). Similarly, small to massive *supraspinatus* tendon tears exhibit increased apoptosis (Benson et al., 2010; Lundgreen et al., 2011). Lastly, the extent of apoptosis positively correlates with increased fiber disorganization in rotator cuff tears (Wu et al., 2011). While insightful, these studies do not implicate apoptosis in the pathogenesis of the disease since the observed increase could solely be a manifestation of the end disease and not part of its mechanistic pathogenesis. However, induced damage positively correlates with increased apoptotic activity and diminished repair response (Andarawis-Puri et al., 2014), as shown by using a previously established *in vivo* rat patellar tendon fatigue-damage model of early-stage tendinopathy (Andarawis-Puri et al., 2012b; Fung et al., 2010).

Diminished repair response in fatigue-damaged tendons could be mitigated by inhibiting apoptosis and, thus, increasing the population of cells available that could repair the damaged matrix. Previous studies suggest that cells in fatigue-damaged tendons likely undergo apoptosis in response to changes in their micromechanical environment that results from extracellular matrix (ECM) damage. The pericellular matrix, which is composed of proteins such as collagen VI and hyaluronan, is a key contributor to the micromechanical environment of the cells, as described in chondrocytes (Guilak et al., 2006). Interestingly, collagen VI and hyaluronan play a protective role against apoptosis and oxidative damage (Cescon et al., 2015; Peters et al., 2011; Presti and Scott, 1994). Furthermore, ECM damage and changed micromechanical environment due to pericellular matrix (PCM) alteration may alter cell fate, *e.g.* cell survival and apoptosis.

Thus, the working hypothesis was that preventing cells in damaged matrix from undergoing apoptosis would prompt cells to stabilize their micromechanical environment through an increase in PCM proteins and promote an increased anabolic ECM production, ultimately leading to recovery of mechanical properties of fatigue-damaged tendons. Given the hypovascular nature of tendons, the first objective was to determine whether apoptosis was preventable using a systemic pan caspase inhibitor (quinolyl-valyl-O-methylaspartyl-[−2, 6-difluorophenoxy]-methyl ketone, Q-VD-OPh). Then, if effective inhibition of apoptosis was achieved, the second objective was to determine whether a larger population of cells produced key ECM and PCM proteins, suggesting stabilization of their microenvironment and leading to improvement in mechanical properties of sub-rupture fatigue-damaged tendons.

## Materials and Methods

Under Institutional Animal Care and Use Committee (IACUC) approval, left patellar tendons (PT) of 74 anesthetized (isoflurane gas: 1-5 % volume, 0.4 L/min) 9-month-old female retired-breeder Sprague-Dawley rats (Charles River Laboratories Inc. and Harlan Laboratories Inc.) were fatigue-loaded as per established protocols (Andarawis-Puri et al., 2012a; Andarawis-Puri et al., 2012b; Andarawis-Puri et al., 2014; Andarawis-Puri and Flatow, 2011; Bell et al., 2015; Fung et al., 2010). Sengupta (2013) suggests that a 9-month female subjected to fatigue load is comparable to a middle 20s human, which is a reasonable time to investigate mechanisms that underlie initiation and pathogenesis of chronic tendinopathies. Briefly, under aseptic conditions, the tibia and patella were exposed, clamped and positioned at ~ 30° flexion. The tibial clamp was secured in a fixed base and the patellar clamp was serially connected to a 222 N load cell (Transducer Techniques, Temecula, CA, USA) and an Instron machine (Instron 8841). Animals were fatigue-loaded for 7200 cycles from 1 to 40 N at 1 Hz. Diagnostic tests were applied prior to (DTI) and post (DT2) fatigue loading by cycling between 1 and 15 N at 1 Hz for 420 or 120 cycles, respectively. Damage parameters (Andarawis-Puri et al., 2012a; Andarawis-Puri et al., 2012b), including hysteresis, loading and unloading stiffness and elongation, calculated from the change between DTI and DT2, confirmed that all groups were similarly damaged. Skin lacerations were sutured with prolene 6-0 sutures (Ethicon) and animals resumed cage activity. Analgesic, 0.06 mg/mL/kg buprenorphine, was injected subcutaneously following fatigue loading and 24 h later. While buprenorphine is not expected to affect cell survival, metabolism or apoptosis (Santiago et al., 2009), any confounding effects from unexpected adverse effects of buprenorphine were mitigated since all animals [both receiving the apoptosis inhibitor and the dimethyl sulfoxide (DMSO) control] received the analgesic. Sham surgeries, wherein the same surgical protocol is utilized for fatigue loading without application of the fatigue loading protocol, are reported in previous studies (Fung et al., 2010) and are found to lead to no differences from naïve tendons.

Prior to fatigue loading, animals were randomly prescribed one of four injection regimens (*n* = 6-8/group): either 2 or 5 d of apoptosis inhibitor Q-VD-OPh (SM Biochemicals LLC, Anaheim, CA, USA) (20 mg/kg/d divided in 2 doses every 12 h; 4 and 10 doses respectively) dissolved in DMSO (14.1 M at >99.5 % purity) or DMSO carrier control delivered by intraperitoneal injection. DMSO-injected fatigue-loaded rats were used as controls to account for the possible cytotoxic effects of DMSO. The first injection was administered at the time of fatigue loading due to the systemic nature of the intraperitoneal injection delivery and the fact that apoptosis likely initiates during the 2 h of fatigue loading (Cardoso et al., 2009). The sample size was determined based on a previous study (Andarawis-Puri et al., 2014) and from an *a priori* analysis to achieve a power of 0.80.

Animals were sacrificed at day 7 or 14 post-fatigue-loading and the quadriceps-patellar-PT-tibia units were harvested, placed under ~ 2 N tension, fixed in Z-fix (Anatech LTD, Battle Creek, MI, USA), decalcified in formic add (Decal Chemical Corporation, Tallman, NY, USA), embedded in paraffin and cut in 6 μm-thick sagittal sections. Apoptosis peaks within the first 2 weeks after fatigue loading (Andarawis-Puri et al., 2014), therefore, the effect of its inhibition on protein production were evaluated within that time course. To address whether apoptosis was preventable using a systemic pan caspase inhibitor, immunohistochemistry (IHC) for cleaved caspase-3 (1 : 1500; Cell Signaling Technologies) was conducted in all groups to determine effectiveness of 2 and 5 d of systemic apoptotic inhibition. To address whether a larger population of cells produced key ECM and PCM proteins, groups where apoptosis was successfully inhibited were stained for ECM proteins [procollagen I (1 : 100; Santa Cruz Biotechnology) and procollagen II (1 : 500; Biobyrt, San Francisco, CA, USA)], PCM proteins [collagen VI (1 : 2000; Abcam) and hyaluronan (1 : 200; MyBiosource, San Diego, CA, USA)] and oxidative stress markers [lysyl oxidase (LOX) (1 : 4000; Abcam) and reactive oxygen species modulator 1 (ROMO1) (1 : 100; Santa Cruz Biotechnology)]. Endogenous peroxidase activity was quenched using 10 % H_2_O_2_ followed by antigen retrieval using DeCal Retrieval Solution for cleaved caspase-3 (Biogenex, Freemont, CA, USA) and proteinase-K (Dako) for all other stains. Non-specific binding was blocked using Rodent Block R for cleaved caspase-3 (Biocare Medical) and Dako Protein Block (Agilent) for all other stains. Rabbit or goat serum without primary antibody were used as negative controls. ImmPACT DAB Substrate kit (Vector Laboratories) was used for chromogen staining. Sections were counterstained with NucRed Dead 647 (Thermo Fisher Scientific) for collagen VI and toluidine blue for all other stains. A blinded user analyzed two sections from each specimen that were 60 μm apart from each other and values were averaged. A total of 6-8 animals per group were used.

200× images of the origin, midsubstance and insertion (tibial end) were analyzed using ImageJ (Schneider et al., 2012). As previously described (Andarawis-Puri et al., 2014; Bell et al., 2015), regions for analysis at the origin and insertion were determined by outlining a trapezoid that bordered the tendon, fibrocartilage and tendon surface. Midsubstance area for analysis was determined by calculating tendon midpoint and capturing two columns of images throughout the full thickness. Negative and positive caspase-3 cells were counted to determine live cell density [reflective of live cells and indicated by cleaved caspase-3 negative cells/area (mm^2^)], apoptotic cell ratio (cleaved caspase-3 positive/total cells, where total cells equal live cells plus apoptotic cells) and total cell density [total cells/area (mm^2^)]. Positive and total procollagen I, procollagen II, collagen VI, hyaluronan, LOX and ROMO1 cells were counted to determine positive cell ratio (positive/total cells).

Mechanical testing was used to determine the effect of inhibition of apoptosis on the mechanical properties of fatigue-damaged tendons. Animals were sacrificed 6 weeks post-fatigue-loading and stored at − 20 °C. Fatigue loading leads to an immediate 20 % stiffness loss that is not recovered out until at least 6 weeks, suggesting that the effect of fatigue loading on the macroscopic mechanical properties would be similar at any time point within this time course (Andarawis-Puri and Flatow, 2011). However, since production of ECM proteins and their incorporation into the matrix can take several weeks (Xue and Jackson, 2015), the evaluation of the mechanical properties was carried out 6 weeks after fatigue loading. At time of testing, the patella-patellar tendon-tibia unit for both fatigue-loaded and contralateral limb was harvested and the tibia was potted in poly(methyl methacrylate) bone cement. Cross-sectional area was calculated by multiplying the average width of three measurements along the length of the tendon by the thickness of the tendon, as measured using a digital caliper with a ~ 2 N load on the tendon. In a phosphate-buffered saline bath, the potted tibia was secured to the base and the patella was clamped. Tendons were loaded with a 2 N preload for 1 min. A stress relaxation test was conducted by ramping to 5 % strain at 5 %/s and held for 300 s. After a 300 s recovery, the tendons were pulled to failure at a rate of 0.3 %/s. A custom Matlab program was used to calculate stiffness and modulus. Cross-sectional area, stiffness, maximum load, modulus and maximum stress were reported.

For IHC analysis, Student’s *t*-tests were utilized to compare the inhibitor and the respective DMSO carrier control group. Inhibitor groups were normalized by their respective DMSO carrier control and non-parametric *t*-tests (Mann-Whitney) were used to compare normalized values over time. For mechanical assessment, the contralateral limbs from each group were first compared by Student’s *t*-test and, subsequently, pooled, since there were no differences. ANOVA with Tukey’s *post-hoc* tests was used to compare DMSO carrier, apoptosis inhibitor and contralateral controls. Significance was set at ^a^ *p* ≤ 0.05. Due to the nature of animal work and biological variability, trends were also reported, which were set to ^b^ *p* ≤ 0.1. Figures depict averages and standard deviations.

## Results

**Fig. 1.**
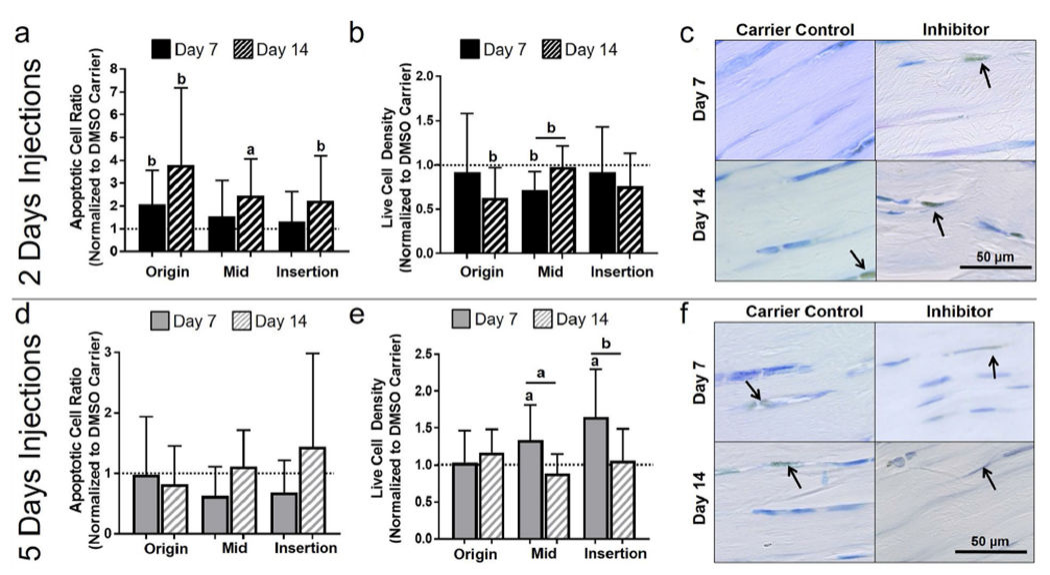
Effect of 2 and 5 days of inhibition of apoptosis on apoptotic activity. With 2 d of injections, (**a**) there was a general increase in apoptotic cell ratio at day 14 and (**b**) a decrease in live cell density at days 7 and 14. (**c**) Representative midsubstance images of cleaved caspase-3 for 2 d of injection at day 7 and 14, showing increased apoptotic (brown) cells for the apoptosis inhibitor at day 14. With 5 d of injection, (**d**) there was no changes in apoptotic cell ratio, however, (**e**) a general increase in live cell density at day 7 was observed. (**f**) Representative midsubstance images of cleaved caspase-3 for 5 d of injection at day 7 and 14, showing increased live (blue) cells for the apoptosis inhibitor at day 7. Arrows show positive staining.

First, whether apoptosis could be effectively inhibited in fatigue-damaged tendons was addressed using a systemic injection of QVD-OPh. Contrary to the working hypothesis, 2 d of apoptosis inhibition increased apoptosis at day 14 in the midsubstance (Fig. 1a). However, supporting the working hypothesis, 5 d of apoptosis inhibition led to an increase in live cell density in the midsubstance and insertion at day 7 (Fig. 1e), which was modulated back to DMSO carrier control levels at day 14. Next, whether a larger population of cells produced key ECM and PCM proteins was addressed and the 5 d injection group, wherein inhibition of apoptosis was effective, was evaluated. Surprisingly, despite a promising initial increase in some regions in procollagen I and collagen VI, inhibition of apoptosis caused an increase in oxidative stress and ultimately led to further deterioration of mechanical properties of fatigue-damaged tendons. The details of these findings are discussed below.

### Dosage response of systemic pan-caspase inhibition on apoptotic activity

#### 2 d injection

Contrary to what was hypothesized, 2 d of apoptosis inhibition increased apoptosis at day 14 in the midsubstance, as measured by apoptotic cell ratio (*p* = 0.05, Fig. 1a) and apoptotic cell density (*p* = 0.05) (Table 1). In addition, a similar trend at day 14 was observed after 2 d of systemic inhibition at the origin (*p* = 0.07) and insertion (*p* = 0.1). Consistent with these findings, 2 d of apoptosis inhibition led to a decrease in live cell density at day 14 in the origin (*p* = 0.07) and at day 7 in the midsubstance (*p* = 0.09). Additionally, apoptosis inhibition decreased live cell density at day 7 as compared to day 14 in the midsubstance (*p* = 0.07).

#### 5 d injection

As hypothesized, 5 d of apoptosis inhibition increased live cells in the midsubstance and insertion (*p* = 0.02 and 0.05, respectively, Fig. 1e and Table 1). This increase in live cell density returned to baseline for both the midsubstance (*p* = 0.02) and insertion (*p* = 0.1) at day 14. There were no significant differences in apoptotic cell ratio between the apoptosis inhibition and DMSO carrier control groups and between day 7 and day 14 (Fig. 1d).

#### 2 d versus 5 d of injection

**Fig. 2.**
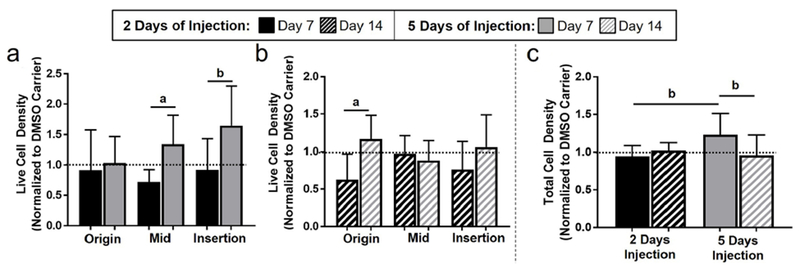
5 days of apoptosis injection increased live cell density. Live cell density was higher for 5 d as compared to 2 d of injection at (**a**) day 7 and at (**b**) day 14. (**c**) 5 d of injection increased total cell density at day 7.

As expected, live cell density was higher at day 7 for 5 d than 2 d of apoptosis inhibition in the midsubstance (*p* = 0.001), with a trend supporting similar findings in the insertion (*p* = 0.07) (Fig. 2 and Table 1). Similarly, at day 14, live cell ratio was higher for 5 d than 2 d of apoptosis inhibition in the origin (*p* = 0.05). Furthermore, there was an increase in total cell density (*p* = 0.01) at day 7 with 5 d *vs.* 2 d of injection. Interestingly, the comparison of 2 and 5 d of DMSO injections showed a decrease in apoptotic cell density in the origin and midsubstance with the longer dosage (Table 1).

### Effects of 5 d of apoptotic inhibition injections on tendon remodeling

#### Mechanical properties

**Fig. 3.**
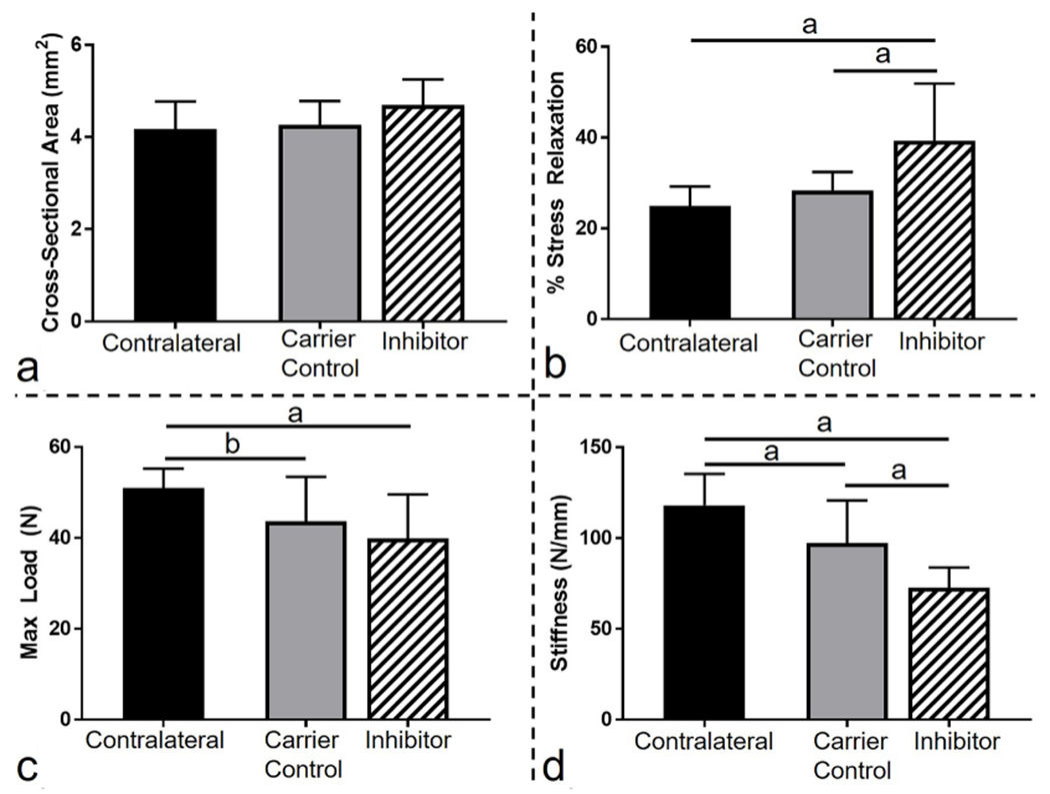
Decrease in mechanical properties caused by inhibition of apoptosis. (**a**) Fatigue loading and subsequent inhibition of apoptosis did not lead to changes in cross-sectional area. (**b**) Inhibition of apoptosis increased stress relaxation (%) as compared to contralateral and carrier control. Fatigue loading led to a decrease in (**c**) max load and (**d**) stiffness. (**d**) Inhibition of apoptosis led to a further decrease in stiffness as compared to carrier control.

Fatigue loading led to the expected decrease in stiffness (*p* = 0.04) and maximum load (*p* = 0.1) for the DMSO carrier control group when compared to the contralateral uninjured control at 6 weeks post-fatigue-loading. Unexpectedly, 5 d of inhibition of apoptosis also led to a decrease in stiffness (*p* < 0.0001) and maximum load (*p* = 0.02) and an increase in percent relaxation (*p* = 0.0006) when compared to the contralateral control. Further comparison between the inhibitor and the carrier control showed that 5 d of apoptosis inhibition significantly decreased the stiffness and increased the percent relaxation (*p* = 0.03) when compared to the DMSO carrier (*p* = 0.02). There was no effect from fatigue loading on the cross-sectional area for either group (*p* = 0.16) (Fig. 3). Consequently, modulus and maximum stress exhibited similar behavior to stiffness and maximum load, respectively (data not shown).

#### Extracellular matrix proteins

**Fig. 4.**
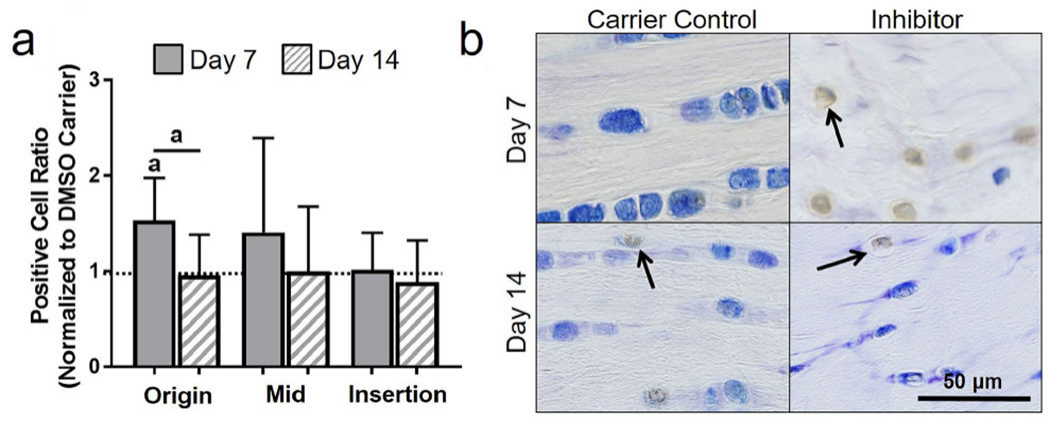
Inhibiting apoptosis increased procollagen I. (**a**) Procollagen I was increased in the origin at day 7. (**b**) Representative origin images of procollagen I for 5 d of injection at day 7 and 14. Arrows show positive staining.

As hypothesized, in the origin, 5 d of apoptosis inhibition led to a higher positive cell ratio for procollagen I at day 7 when compared to day 14 (*p* = 0.03) and to DMSO control (*p* = 0.05) (Fig. 4). Unexpectedly, there was no significant effect of apoptosis inhibition on procollagen I in either the midsubstance or the insertion at day 7 or 14. To provide further context, a representative lower magnification image of procollagen I is shown in Fig. 10a.

**Fig. 5.**
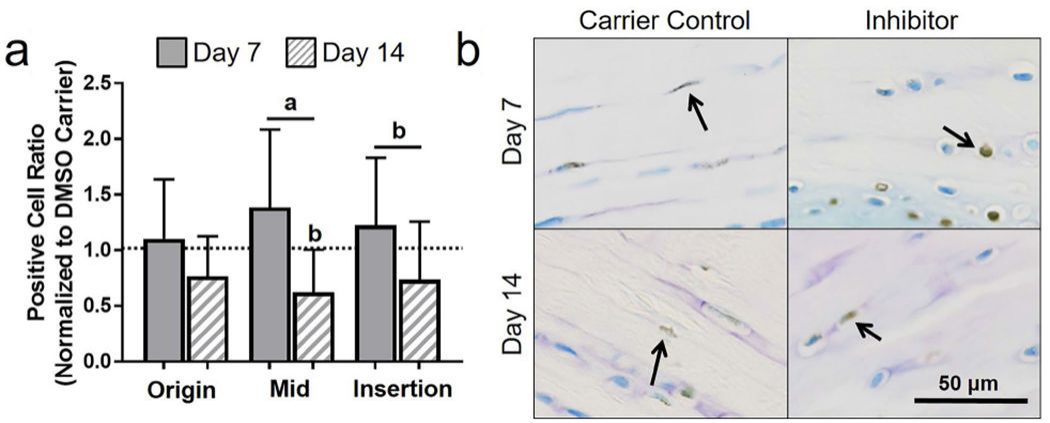
Inhibiting apoptosis increased procollagen II. (**a**) Procollagen II was generally increased at day 7 as compared to day 14. (**b**) Representative insertion images of procollagen II for 5 d of injection at day 7 and day 14. Arrows show positive staining.

Procollagen II was modulated by inhibition of apoptosis in the midsubstance only. More specifically, apoptosis inhibition decreased procollagen-II-positive cell ratio in the midsubstance as compared to DMSO carrier (*p* = 0.1) at day 14. A lower positive cell ratio for procollagen II was found for the 5 d inhibition group at day 14 in comparison to day 7 in the origin (*p* = 0.05) and insertion (*p* = 0.1) (Fig. 5). To provide further context, a representative lower magnification image of procollagen II is shown in Fig. 10b.

**Fig. 6.**
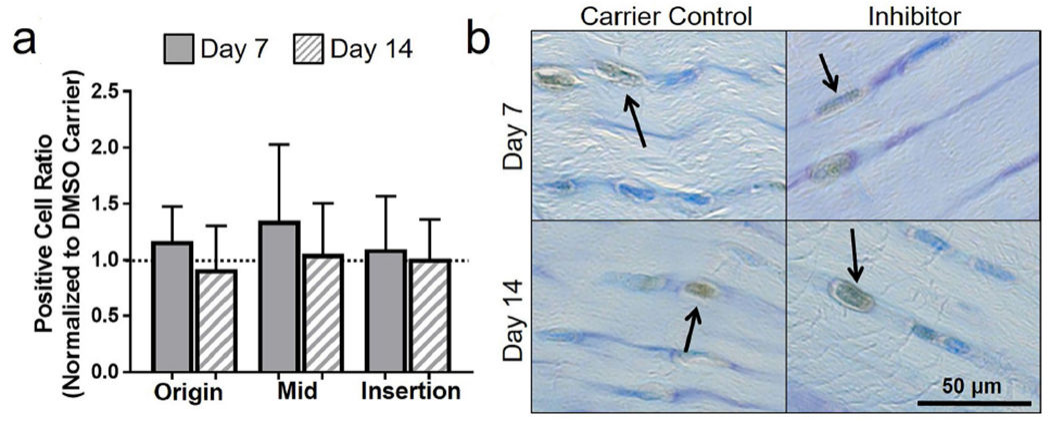
Inhibiting apoptosis caused no changes in LOX. (**a**) Inhibition of apoptosis in fatigue-damaged tendons had no effect on LOX. (**b**) Representative insertion images of LOX for 5 d of injection at day 7 and day 14. Arrows show positive staining.

Unexpectedly, inhibition of apoptosis had no effect on LOX, an ECM cross-linker, in the origin, midsubstance or insertion (Fig. 6). To provide further context, a representative lower magnification image of LOX is shown in Fig. 10c.

#### Pericellular matrix proteins

**Fig. 7.**
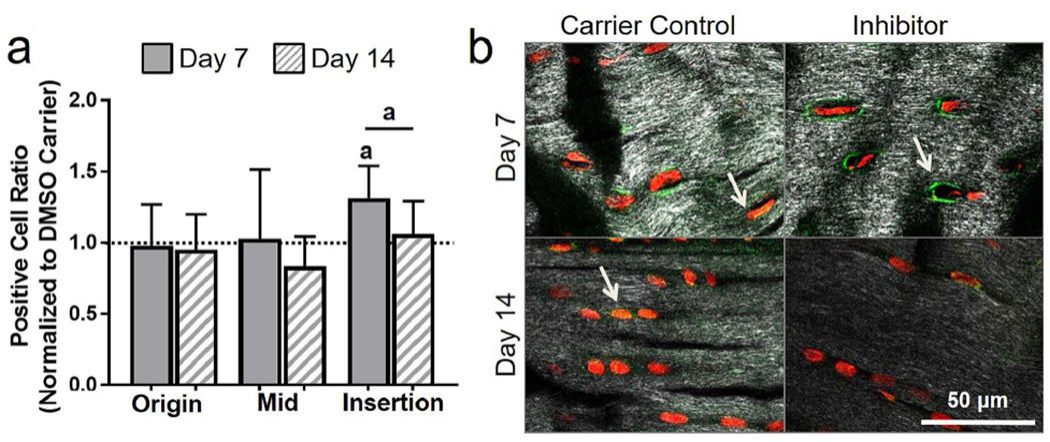
Inhibiting apoptosis increased collagen VI. (**a**) Collagen VI was increased in the insertion at day 7. (**b**) Representative insertion images of collagen VI for 5 d of injection at day 7 and day 14. Arrows showing positive staining.

Surprisingly, apoptosis inhibition had no effects on collagen VI in the origin or midsubstance. However, as hypothesized, 5 d of apoptosis inhibition led to an increase in collagen-VI-positive cell ratio at day 7 as compared to DMSO carrier (*p* = 0.05) and to the effect of the inhibitor at day 14 (*p* = 0.05) (Fig. 7). To provide further context, a representative lower magnification image of collagen VI is shown in Fig. 10d.

**Fig. 8.**
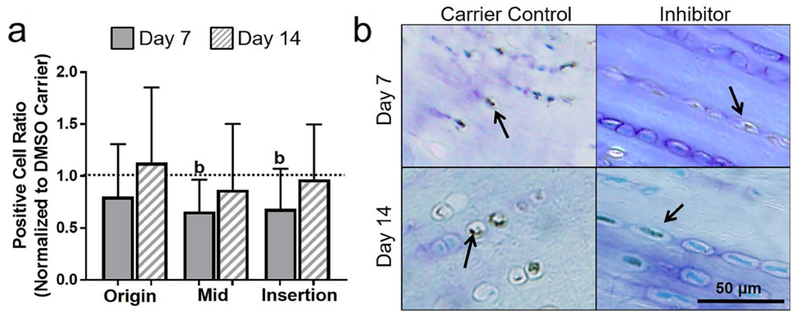
Inhibiting apoptosis decreased hyaluronan. (**a**) Hyaluronan was generally decreased at day 7. (**b**) Representative insertion images of hyaluronan for 5 d of injection at day 7 and day 14. Arrows show positive staining.

Contrary to the working hypothesis, apoptosis inhibition had no effect on hyaluronan at the origin. In addition, apoptosis inhibition led to an unexpected decrease in hyaluronan-positive cell ratio in the midsubstance (*p* = 0.1) and insertion (*p* = 0.1) (Fig. 8). To provide further context, a representative lower magnification image of hyaluronan is shown in Fig. 10e.

#### Cellular stress markers

**Fig. 9.**
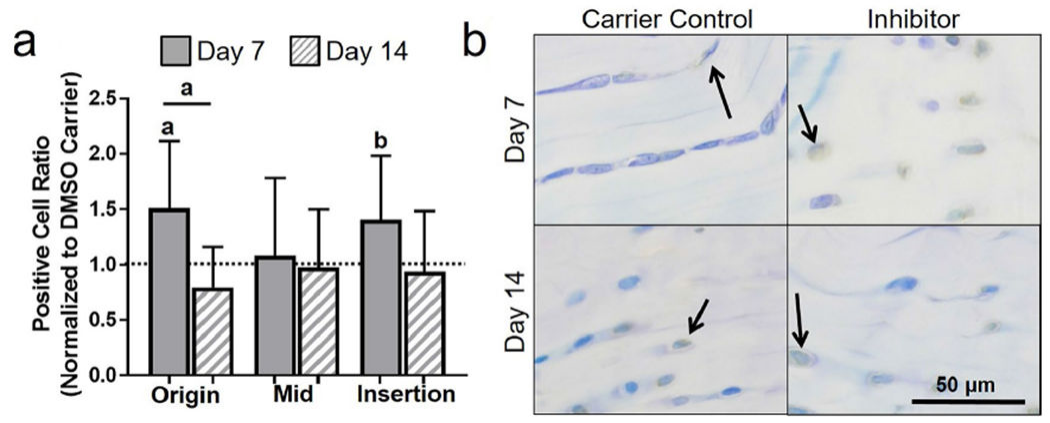
Inhibiting apoptosis increased ROMO1. (**a**) ROMO1 was generally increased at day 7. (**b**) Representative origin images of hyaluronan for 5 d of injection at day 7 and day 14. Arrows show positive staining.

**Fig. 10.**
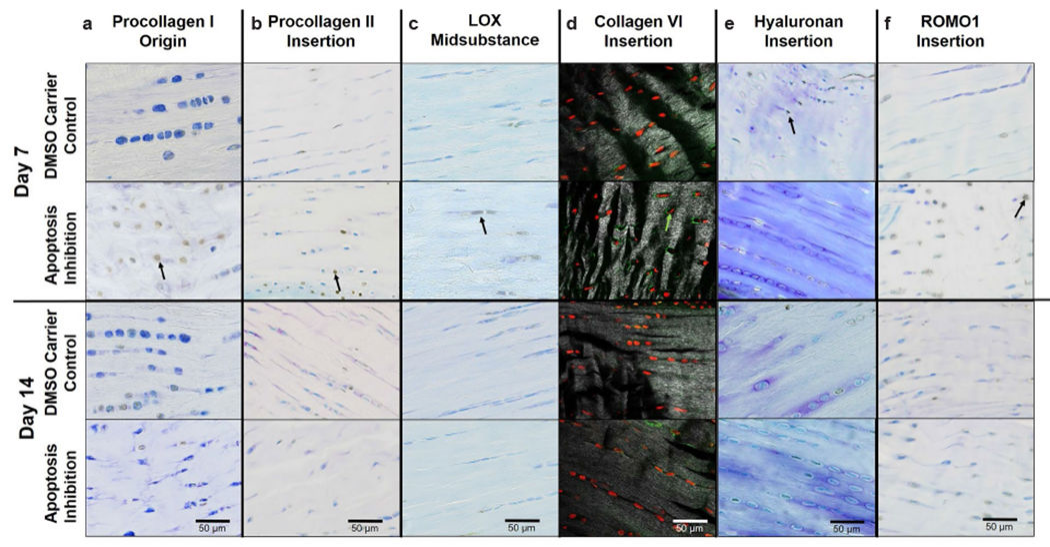
Overall effect of inhibiting apoptosis on key proteins. Representative images of 5 d injection at day 7 or 14 showing (**a**) increased procollagen I for the apoptosis inhibitor at day 7, (**b**) increased procollagen II for the apoptosis inhibitor at day 7, (**c**) no changes in LOX, (**d**) increased collagen VI for the apoptosis inhibitor at day 7, (**e**) decreased hyaluronan for the apoptosis inhibitor at day 7 and (**f**) increased ROMO1 for the apoptosis inhibitor at day 7. Arrows show positive staining.

Contrary to the working hypothesis, in the origin, inhibition of apoptosis led to an increase in ROMO1-positive cell ratio at day 7 when compared to DMSO carrier (*p* = 0.05) and to the effect of the inhibitor at day 14 (*p* = 0.03). Similarly, in the insertion, apoptosis inhibition increased ROMO1-positive cell ratio at day 7 as compared to DMSO carrier control (*p* = 0.06). Inhibition of apoptosis had no effect on ROMO1 in the midsubstance (Fig. 9). To provide further context, a representative lower magnification image of ROMO1 is shown in Fig. 10f.

## Discussion

While clinical biopsies of late stage tendinopathy show increased apoptotic activity (Lian et al., 2007), those findings do not demonstrate a role for apoptosis in pathogenesis of tendinopathy. Previous studies show a direct correlation between severity of damage-induced, increased apoptotic activity and inhibition of remodeling genes, suggesting that apoptosis may be implicated in the pathogenesis of tendinopathy (Andarawis-Puri et al., 2012b; Andarawis-Puri et al., 2014). The working hypothesis was that non-damaged cells in fatigue-damaged tendons likely undergo apoptosis because of their altered loading environment that results from the matrix damage. Thus, the premise of the current study was that preventing cells in damaged matrix from undergoing apoptosis would prompt them to modulate their microenvironment to promote survival and ultimately promote matrix repair. In the present study, the effect of apoptosis inhibition on the ratio of cells positive for PCM proteins (indicative of changes into the microenvironment of the cells) and ECM (indicative of remodeling) and the resulting mechanical properties were evaluated. Two short durations of systemic pan caspase inhibitor [shorter (2 d) and longer (5 d)] were used to mitigate apoptotic activity, as it was expected that complete inhibition of apoptosis, as might be achieved by very long durations, might also have detrimental effects. The apoptosis inhibitor was dissolved in DMSO carrier and consequently, the effect of the inhibitor on fatigue-loaded tendons was determined through comparison with fatigue-loaded animals that received the DMSO carrier control. While DMSO use results in cytotoxicity (Galvao et al., 2014), the used concentration of DMSO was based on a previous study of apoptosis inhibition in bone, brain, spinal cord and heart (Keoni and Brown, 2015), suggesting a limited confounding effect of DMSO on cell death. Interestingly, comparison of the effect of 2 *versus* 5 d of DMSO injections in fatigue-damaged tendons showed a surprising general decrease in apoptosis with the longer period of DMSO injection. While unexpected, a study by Banič et al. (2011) on cells in culture shows that DMSO can contribute to prolonged inactivation of the apoptosis pathways. Alternatively, the decrease in apoptosis (or increase in live cells) associated with 5 d *versus* 2 d of injections could be a feedback loop in response to an earlier rise in apoptosis. Regardless, normalizing the effect of the apoptotic inhibitor (in DMSO carrier) to the DMSO on fatigue-loaded control took into account the effect of DMSO, allowing for isolation of the effect of the inhibitor.

The longer dosage of inhibitor more effectively inhibited apoptosis, as indicated by increased live cell density at day 7, particularly in the midsubstance and insertion. Additionally, since there was no change in apoptotic cell ratio, the results suggested that the longer dosage of the inhibitor was both inhibiting apoptosis and promoting proliferation or cell recruitment. This was consistent with a study by Hua et al. (2015) showing that inhibition of caspase-3 pathways modulates cell cycles and can prompt an increase in the number of cells entering S-phase, ultimately leading to an increase in proliferation. In contrast, the shorter duration of apoptosis inhibitor caused a delayed and enhanced apoptotic response 14 d after chronic injury, suggesting that very short durations were ineffective at allowing cells to modify their microenvironment to mitigate the effect of the damaged matrix.

Effective inhibition of apoptosis was manifested as an increase in live cell density in the midsubstance and insertion. Interestingly, inhibition of apoptosis led to an increase in procollagen I in the origin at day 7 despite the absence of any increase in live cell density in this region. Since a pan caspase inhibitor was utilized, it was possible that cells that were not yet committed to undergo apoptosis (as indicated by caspase-3) underwent inhibition of other caspases that are earlier in the apoptosis pathway. These cells were likely still in a stressed state, as indicated by the accompanying increase in ROMO1, prompting synthesis of collagen I to stabilize their surrounding damaged ECM. Since collagen I is essential for imparting the tendon with its capacity to bear tensile loads (Screen et al., 2015), this observed increase was expected to ultimately lead to improved mechanical properties. Furthermore, effective inhibition of apoptosis led to a decrease in procollagen II in the midsubstance at day 14. Collagen II is a main constituent of tissues that predominantly bear compressive loads and its increase in the midsubstance is typically associated with degeneration (Waggett et al., 1998). Thus, the observed decrease in procollagen II associated with inhibition of apoptosis was initially interpreted as an indicator of ECM repair. However, despite these early changes in ECM protein synthesis that were consistent with remodeling of ECM damage, inhibition of apoptosis was found to lead ultimately to further deterioration of mechanical properties 6 weeks post-fatigue-loading.

There are several possible explanations for the absence of an improvement in mechanical properties after inhibition of apoptosis, despite the observed modulation in procollagen I and II. A decrease in procollagen II in the midsubstance might not be sufficient to overcome the effect of the damage in the insertion. Similarly, procollagen I was increased only in the origin, suggesting that the effect of the damage in the midsubstance and the highly damaged insertion region could overwhelm the observed remodeling in the origin. Furthermore, increased procollagen I might not lead to effective incorporation of collagen I into the matrix. This notion was further supported by the lack of change in LOX, a protein that plays an essential role in collagen fibril formation and ECM crosslinking (Herchenhan et al., 2015; Smith-Mungo and Kagan, 1998). Consequently, the lack of modulation of LOX from apoptosis inhibition suggested no new collagen formation.

Studies on cartilage (Fehrenbacher et al., 2003; Li et al., 2013; McCulloch et al., 2014) motivated the hypothesis that cells in regions of damaged tendon ECM will form an enhanced PCM to maintain mechanical stimulation, thereby reducing cell stress and protecting against apoptosis. Interestingly, the results of this study show an increase in collagen VI that was not associated with any increase in procollagen I or any other change that could be indicative of a remodeling attempt. In fact, ROMO1, a stress marker, was also concurrently increased in the insertion region. Taken together, these data suggested that the excessive ECM damage in the insertion region, along with the complex loading environment, could have prompted an increase in collagen VI, which was ineffective at normalizing the microenvironment of the cells. Collagen VI is typically associated with regions that experience complex loads, such as the insertion (Waggett et al., 1998). Thus, other PCM proteins, such as hyaluronan, were expected to be more critical in the tensile load-bearing midsubstance. Surprisingly, hyaluronan was decreased in the midsubstance and insertion. While unexpected, Yamazaki et al. (2003) show that cyclic stretch of chondrocytes in cartilage leads to an increase in reactive oxygen species (ROS) that depolymerize hyaluronan, providing a possible explanation for the observed decrease in hyaluronan.

There are several possible explanations for the deterioration in mechanical properties of fatigue-damaged tendons that resulted from inhibition of apoptosis. Prevention of apoptosis through the caspase pathways could prompt over compensation of other pathways, such as programmed cell necrosis through the autophagy pathway (Zhao et al., 2015). Consistent with the results of the current study, an increased ROS production is reported. Studies of intervertebral disc implicate ROS production in disc degeneration through a catabolic effect *via* mitogen-activated protein kinases (MAPKs) signaling and increase matrix metalloproteinases activity (Li et al., 2012; Suzuki et al., 2015). A similar mechanism might be implicated in fatigue-damaged tendons.

Cell-ECM interactions, through agents such as integrins, connexins and cadherins, regulate cell homeostasis by communicating chemical, structural and mechanical cues between the cell and the surrounding ECM environment. Disruptions to such interactions may alter cell signaling, leading to either a reparative (anabolic) or pathological (catabolic) effect by altering cell metabolism (for example, protein production) and/or cell fate (for example, proliferation, differentiation or apoptosis) (Humphrey et al., 2014; Qi et al., 2011). The current study showed that apoptosis inhibition enabled tenocytes to produce matrix proteins but the transient effects at the origin and insertion suggested that production of more matrix proteins was required and/or that cell-ECM interactions needed to be restored. Combining apoptosis inhibition with another therapeutic that restores cell-ECM interactions, such as physiologic mechanical loading (Popov et al., 2015) or exercise, might be optimal for long-term cell survival and tissue repair.

One limitation of this study was that the pan-caspase inhibitor was systemically administered, therefore, the amount of inhibitor that was taken up by each region of the tendon could not be directly assessed. For instance, vascularity, which typically varies among the insertion, origin and midsubstance (Pufe et al., 2005), might be further altered non-uniformly by fatigue loading, impacting the capacity of each region to uptake the drug. While this limitation did not affect the conclusions drawn in the current study, a local apoptosis delivery method and a full dosage-response study would provide further insight. Another limitation was that it was not possible to track which cells were inhibited from undergoing apoptosis, to determine how their protein production was altered. Future studies will utilize multiple staining techniques to assess levels of several proteins for the same cells at a given timepoint. Lastly, while previous studies show a correlation between ECM damage and apoptosis (Andarawis-Puri N et al., 2014), the nature of the altered loading environment associated with ECM damage that prompts apoptosis remains unknown and should be evaluated in future studies. For instance, cells residing in severely damage ECM are likely under-loaded due to loss of cell-ECM interactions. Conversely, prior to reaching the under-loaded state, cells in damaged ECM were likely overloaded due to plastic deformation of collagen fibers. Understanding the loading state that prompts apoptosis would provide further insight into the mechanotransduction pathways that are associated with aberrant micro-mechanical cellular environments.

In conclusion, inhibiting apoptosis, thereby forcing cells to stay alive in the presence of ECM damage, led to further degeneration, despite some early indicators of remodeling. While inhibition of apoptosis might still be an effective approach for promoting repair of fatigue-damaged tendons, a different approach, wherein cell survival is the outcome of a modulated micro-environment of the cells should be considered.

## Figures and Tables

**Table 1.** Cell densities for DMSO carrier control and apoptosis inhibitor injection protocols. Cell densities for 2 and 5 d of DMSO carrier control or apoptosis inhibitor injection at day 7 or 14. Shared uppercase letters indicate significant differences (*p* ≤ 0.5). Shared lowercase letters indicate trends (*p* ≤ 0.1).

2 d injection			Origin	Midsubstance	Insertion	5 d injection			Origin	Midsubstance	Insertion
**Apoptotic cell density (cells/mm^2^)**	**7 d**	DMSO	127.4 ± 54.5^*C*^	136.5 ± 116.8^*d*^	272.3 ± 200.6	**Apoptotic cell density (cells/mm^2^)**	**7 d**	DMSO	53.5 ± 54.l^*C*^	40.8 ± 67.4^*d*^	121.3 ± 194.4
		Drug	250.6 ± 286.4^*e*^	171.5 ± 204.0	292.9 ± 272.6^*f*^			Drug	39.4 ± 44.3^*e*^	32.3 ± 25.1	91.3 ± 76.6^*f*^
	**14 d**	DMSO	69.2 ± 28.5^*a*^	51.5 ± 28.3^*B*^	112.8 ± 58.2^*g*^		**14 d**	DMSO	51.5 ± 26.6	40.6 ± 29.5	53.8 ± 57.1^*g*^
		Drug	250.1 ± 243.6^*aH*^	122.4 ± 75.1^*BI*^	229.6 ± 226.3			Drug	47.8 ± 37.5^*H*^	40.9 ± 30.4^*I*^	125.5 ± 125.7
**Live cell density (cells/mm^2^)**	**7 d**	DMSO	586.1 ± 133.9	737.3 ± 244.8^*jN*^	948.6 ± 345.3	**Live cell density (cells/mm^2^)**	**7 d**	DMSO	563.3 ± 204.8	471.8 ± 106.9^*LN*^	853.6 ± 129.1^*M*^
		Drug	536.9 ± 388.4	531.8 ± 149.8^*jP*^	870.7 ± 487.2			Drug	557.9 ± 271.6	782.6 ± 248.9^*LP*^	1182.8 ± 326.9^*M*^
	**14 d**	DMSO	809.6 ± 241.6^*k*^	599.8 ± 248.9	1169.9 ± 353.5		**114 d**	DMSO	638.5 ± 203.8	639.6 ± 137.9	1078.2 ± 105.2
		Drug	508.3 ± 276.1^*kq*^	583.9 ± 144.2	888.6 ± 440.4			Drug	745.5 ± 201.0^*q*^	564.9 ± 169.0	1141.6 ± 464.3
**Total cell density (cells/mm^2^)**	**7 d**	DMSO	724.0 ± 147.3	873.8 ± 320.7^*T*^	1223.7 ± 404.7	**Total cell density (cells/mm^2^)**	**7 d**	DMSO	616.8 ± 202.1	512.6 ± 102.9^*RT*^	981.4 ± 176.3^*s*^
		Drug	793.4 ± 306.8	703.4 ± 143.2	1163.7 ± 249.3			Drug	598.4 ± 268.4	814.9 ± 236.3^*R*^	1254.1 ± 330.3^*s*^
	**114 d**	DMSO	878.8 ± 230.9	651.4 ± 270.3	1282.7 ± 390.2		**14 d**	DMSO	690.1 ± 221.8	680.2 ± 136.6	1134.1 ± 99.6
		Drug	758.3 ± 250.6	706.4 ± 123.6	1121.1 ± 414.4			Drug	795.7 ± 190.2	605.9 ± 188.8	1267.7 ± 387.8

## Discussion with Reviewer

**Keri Gardner:** Changes in collagen and pro collagens may be due to increased collagenases because of microscopic damage (Lavagnino et al., 2006 additional reference). What is the authors opinion about it?

**Authors:** The changes in collagen levels could be caused by local collagenase due to the fatigue-damaged tendons. However, this phenomenon would be equally likely to occur in both the apoptosis inhibitor group and the DMSO carrier control group. Thus, any observed differences between the apoptosis inhibitor and DMSO control groups in the procollagens/collagens could only be attributed to the effect of blocking apoptosis.
